# Inferring ancient metabolism using ancestral core metabolic models of enterobacteria

**DOI:** 10.1186/1752-0509-7-46

**Published:** 2013-06-11

**Authors:** David J Baumler, Bing Ma, Jennifer L Reed, Nicole T Perna

**Affiliations:** 1 Genome Center of Wisconsin, University of Wisconsin-Madison, Madison, Wisconsin, USA; 2 Department of Chemical and Biological Engineering, University of Wisconsin-Madison, Madison, USA; 3 Department of Genetics, University of Wisconsin-Madison, Madison, USA; 4 Current affiliation: Institute for Genome Sciences, University of Maryland School of Medicine, Baltimore, MD 21201, USA

**Keywords:** Constraint-based modeling, Enterobacteria, Metabolic network reconstruction, Ancient metabolism, Paleo systems biology, Ancestral core

## Abstract

**Background:**

*Enterobacteriaceae* diversified from an ancestral lineage ~300-500 million years ago (mya) into a wide variety of free-living and host-associated lifestyles. Nutrient availability varies across niches, and evolution of metabolic networks likely played a key role in adaptation.

**Results:**

Here we use a paleo systems biology approach to reconstruct and model metabolic networks of ancestral nodes of the enterobacteria phylogeny to investigate metabolism of ancient microorganisms and evolution of the networks. Specifically, we identified orthologous genes across genomes of 72 free-living enterobacteria (16 genera), and constructed core metabolic networks capturing conserved components for ancestral lineages leading to *E. coli/Shigella* (~10 mya), *E. coli/Shigella/Salmonella* (~100 mya), and all enterobacteria (~300-500 mya). Using these models we analyzed the capacity for carbon, nitrogen, phosphorous, sulfur, and iron utilization in aerobic and anaerobic conditions, identified conserved and differentiating catabolic phenotypes, and validated predictions by comparison to experimental data from extant organisms.

**Conclusions:**

This is a novel approach using quantitative ancestral models to study metabolic network evolution and may be useful for identification of new targets to control infectious diseases caused by enterobacteria.

## Background

Initially named for a group of intestinal bacteria, members of the family *Enterobacteriaceae* are distributed worldwide and are found in soil, water, agronomic crops and produce, plants and trees, and in animals ranging from insects to humans. Pathogenic enterobacteria cause biomedically and agriculturally significant diseases, and historically have resulted in numerous pandemics, foodborne outbreaks, and nosocomial infections, arguably impacting human health more than any other microbial family. Enterobacteria have been extensively studied in the laboratory due to their importance to human health and as standard laboratory strains for molecular biology. The family includes 44 distinct genera and 176 named species [1], and there are over 150 complete or nearly complete genomes currently available for enterobacteria. Extensive comparative analysis between these genomes has revealed some of the genomic variations linked to host/niche specialization. The metabolic gene content of these genomes is complex, with each strain predicted to contain over 800 genes encoding metabolic enzymes and transporters. One method to investigate the complexity of genome-scale metabolic networks is through the construction of computational models.

Computational modeling of bacterial metabolism offers a promising approach to predict strain-to-strain variation in metabolic capabilities and microbial strategies used in different environments, including host tissues. The number of available genome-scale metabolic models (GEMs) has grown in the last ten years to over 50 GEMs, and they capture the metabolic capabilities of numerous microbial taxa important to human health, biotechnology and bioengineering [2,3]. Systems biology combines computational and experimental approaches to study the complexity of biological networks at a systems level, where the cellular components and their interactions lead to complex cellular behaviors. Genome-scale biological networks have proven useful for interpreting high-throughput data and generating computational models. Mathematical models are constructed from network reconstructions, and they include variables, parameters, and equations to describe the potential behavior of these networks. Starting with *E. coli* K-12 numerous types of genome-scale biological networks have been constructed including metabolic, regulatory, and transcriptional and translational machinery [4-9], and additional GEMs for additional enterobacteria have recently been constructed [4,10-15].

To date, GEMs of enterobacteria have been constructed for three standard laboratory *E. coli* strains [4,6-8,10], four pathogenic *E. coli* strains [4], one *Salmonella* strain [14,16], one *Klebsiella* strain [12], two *Yersinia* strains [10,13], and one insect endosymbiont, *Buchnera*[15]. These GEMs have been used to bioengineer strains for valuable end product formation [17-22], to conduct simulations to investigate metabolic processes during host-pathogen interactions [14], to identify differentiating metabolic properties between commensal and pathogenic *E. coli* strains [4], and to provide insight into the genome evolution of other enterobacteria [23-25]. In addition to strain-specific enterobacterial GEMs, recently 16 *E. coli* genomes were used to construct models from the combined genomic content of these *E. coli* strains, representing the intersection (ancestral core) and union (pangenome) and revealed new insight into the evolution of this species [4].

Members of the family *Enterobacteriaceae* diversified from a common ancestor ~300-500 million years ago (mya) into a wide variety of free-living and host-associated lifestyles [26,27], yet based on conserved metabolic phenotypes of all modern enterobacteria, little is known about ancestral traits of metabolism beyond that they were able to catabolize glucose and grow in the presence or absence of oxygen [1]. Here the metabolism of ancient microorganisms has been investigated by identifying orthologous genes shared in the genomes of 72 free-living enterobacteria from 16 genera, and constructing metabolic networks representing the ancestral core at three phylogenetic points: the *E. coli/Shigella* ancestral core (~10 mya), the *E. coli/Shigella/Salmonella* ancestral core (~100 mya), and the enterobacterial ancestral core (~300-500 mya). Using these metabolic models we have analyzed the metabolic capacity for carbon, nitrogen, phosphorous, sulfur, and iron utilization in aerobic and anaerobic conditions and have identified conserved and differentiating catabolic phenotypes and validated these predictions by comparison to experimental data. Apart from our previous publication on *E. coli*, this is the first study to use constraint-based modeling to examine the metabolic properties of ancestral bacteria and provides new insight into the evolution of metabolism for the family *Enterobacteriaceae*.

## Results

The first GEM for *E. coli* K-12 MG1655, was developed 10 years ago and has undergone numerous improvements and updates. It is now a sophisticated compartmentalized model containing over 1,300 genes and 2,400 reactions [4,7]. It has been used extensively for biotechnology, discovery applications, and to study evolutionarily related enterobacteria. Here we generated ancestral core metabolic GEMs at three phylogenetic branching points within the family *Enterobacteriaceae* from a *E. coli* K-12 MG1655 GEM [4] based on the retained metabolic capability determined through a comparative genomic analysis of 72 enterobacterial genomes. We validated these models by comparing *in silico* carbon source utilization predictions to experimental data spanning 36 extant strains from 16 genera to examine the shared metabolic capabilities of modern-day enterobacteria and the impact of changes in the metabolic network on phenotypic traits.

### Phylogenetic reconstruction for the family *Enterobacteriaceae*

**Figure 1:**
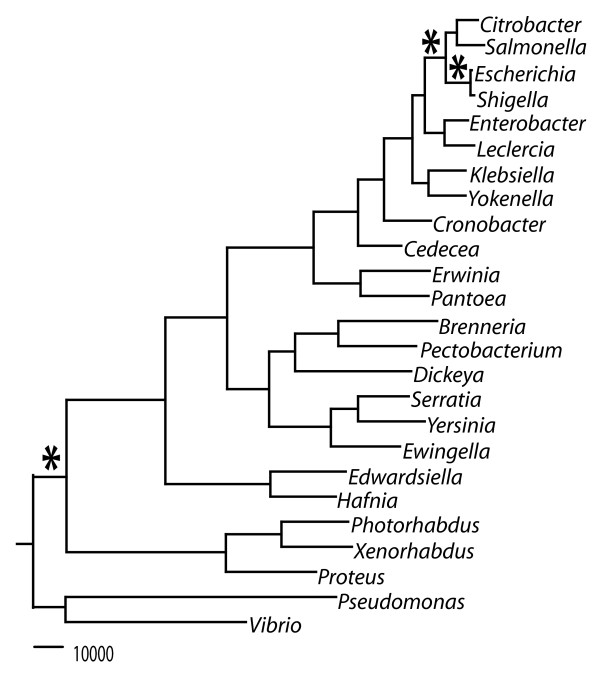
**Phylogenetic reconstruction for the family of *Enterobacteriaceae*.** Total evidence Maximum Likelihood tree (* indicates phylogenetic branching point for ancestral core metabolic models).

A total evidence tree was constructed for the enterobacteria with available genome sequence data (Figure 1). The total evidence tree is extremely robust with full support at every internal tree node. Trees were concordant between the neighbor joining and maximum likelihood methods, as well as between the total evidence trees and consensus trees. This phylogeny in phylogram form indicates a general trend that plant-associated clades have a relatively deeper split including both the soft-rotting clade with *Dickeya* and *Pectobacterium,* as well as the *Erwinia-Pantoea* clade, suggesting ancient speciation events for these genera. The clade including *Escherichia*, *Salmonella* and other animal-associated organisms shows relatively shorter intra-clade branches length, indicating their relatively recent speciation. This phylogenetic tree for the enterobacteria was used to determine the order of genomes used in subsequent analysis for ancestral core metabolic gene determination.

### Generation of an *E. coli/Shigella* core metabolic network

**Figure 2:**
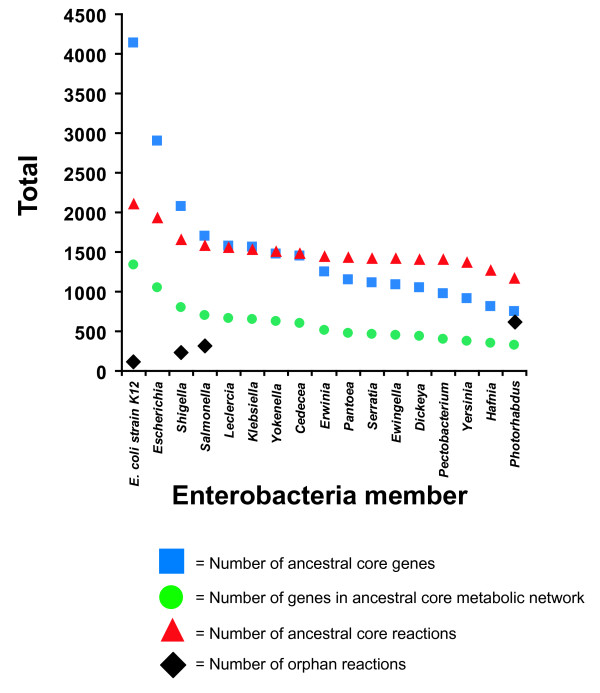
***Enterobacterial *core evolution according to the number of sequenced genomes listed in Table**5**.**

**Figure 3:**
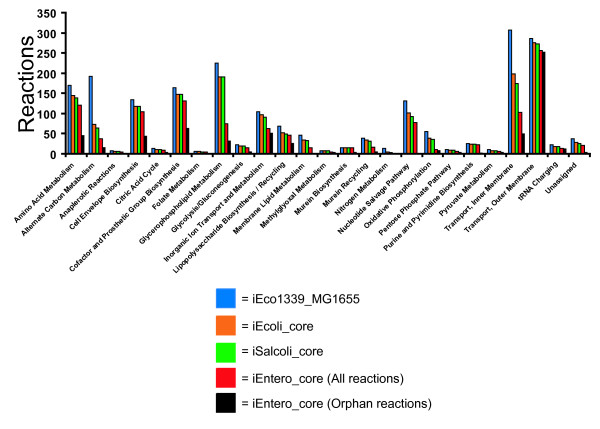
***E. coli/Shigella*, *E. coli*/*Shigella*/*Salmonella*, and enterobacterial ancestral core metabolic model composition and orphan reactions contained in the enterobacterial ancestral core metabolic model classified into subsystems.**

*E. coli* and *Shigella* strains are thought to have diverged from a common ancestor ~10 mya [27]. Although this is the most recent ancestral state we investigate here, gene losses and acquisition of genes via horizontal transfer have led to extensive differences in genome content among descendants of this node with some *E. coli* strains differing by as much as 25% of their gene content [28]. It is of interest to understand the extent to which this has impacted the metabolic network over this time frame. We assume that genes conserved across all strains represent a conservative estimate of the core genome of the ancestor of modern *E. coli* strains. From the 16 *E. coli* and seven *Shigella* spp. genomes we identified a collection of 2,073 conserved genes to construct iEcoli_core (Figure 2). Of these conserved genes, 790 have been experimentally characterized for metabolic function and are present in the *E. coli* K-12 MG1655 GEM (iEco1339_MG1655) [4]. As previously described for construction of ancestral core metabolic models [4], a metabolic network for the *E. coli/Shigella* ancestral core was made by removing reactions from the iEco1339_MG1655 network if orthologs to the associated gene(s) were absent in one or more of the 23 genomes and if the reactions did not have any isozymes. If removing a reaction prevented biomass production (predicted using flux balance analysis) then the reaction was added back to the metabolic model without a gene associated with it and the reaction was classified as an orphan reaction (i.e. a reaction without any associated genes). Using this approach 549 ORFS associated with 454 reactions were removed from iEco1339_MG1655 resulting in an *E. coli* core metabolic network (iEcoli_core) consisting of a total of 790 ORFs and 1,674 reactions (Table 1), and the reactions retained in the core model were classified based on metabolic subsystem (Figure 3).

**Table 1:** Metabolic model information and reaction subsystem classification

**Model**	**iEco1339_MG1655**	**iEcoli_core**	**iSalcoli_core**	**iEntero_core**
Genomes included in analysis	1	23	39	72
Genes	1339	790	683	325
Reactions Total	2,128	1,674	1,601	1,191
Orphan Reactions	100	207	272	677
Reactions by subsystem				
Alternate Carbon Metabolism	192	73	64	37
Amino Acid Metabolism	170	144	139	121
Anaplerotic Reactions	8	6	6	4
Carnitine Degradation	1	0	0	0
Cell Envelope Biosynthesis	134	118	118	104
Citric Acid Cycle	13	10	10	9
Cofactor and Prosthetic Group Biosynthesis	164	148	147	131
Folate Metabolism	6	6	5	4
Glycerophospholipid Metabolism	225	191	191	75
Glycine Betaine Biosynthesis	1	1	1	1
Glycolysis/Gluconeogenesis	22	20	19	15
Glyoxylate Metabolism	4	2	2	2
Inorganic Ion Transport and Metabolism	105	97	91	63
Lipopolysaccharide Biosynthesis / Recycling	68	52	49	46
Membrane Lipid Metabolism	46	34	33	15
Methylglyoxal Metabolism	8	7	7	5
Murein Biosynthesis	15	15	15	15
Murein Recycling	38	34	31	17
Nitrogen Metabolism	13	4	3	0
Nucleotide Salvage Pathway	131	101	92	77
Oxidative Phosphorylation	55	39	36	10
Pentose Phosphate Pathway	10	9	9	6
Purine and Pyrimidine Biosynthesis	26	24	24	22
Pyruvate Metabolism	10	8	7	6
Transport, Inner Membrane	307	198	174	103
Transport, Outer Membrane	39	28	25	9
Transport, Outer Membrane Porin	247	247	247	247
tRNA Charging	22	18	18	14
Unassigned	37	28	26	21

### Generation of an *E. coli/Shigella/Salmonella* core metabolic network

*E. coli* and *Salmonella* strains are thought to have diverged from a common ancestor ~100 mya [27] and it is of interest to understand how the metabolic networks have evolved over time to have an estimate of the metabolic capabilities of an ancestor to modern day *E. coli* and *Salmonella* strains. We assume that genes conserved across all strains represent a conservative estimate of the core genome of the ancestor of modern *E. coli* and *Salmonella* strains and from these 16 *E. coli*, seven *Shigella* spp., and 16 *Salmonella* spp. genomes the collection of 1,703 conserved genes were used to construct the ancestral metabolic model iSalcoli_core (Figure 2). There are 683 of these genes that have characterized metabolic function in the *E. coli* K-12 MG1655 GEM (iEco1339_MG1655) [4]. A metabolic network for the *E. coli/Shigella/Salmonella* ancestral core was made as before by removing reactions from the iEco1339_MG1655 network if orthologs for the associated genes were absent in one or more of the 39 genomes. Reactions were added back as orphan reactions if they were essential for biomass production during growth simulation in minimal media with glucose as the sole carbon source. Using this approach 657 ORFS associated with 527 reactions were removed from iEco1339_MG1655 resulting in an *E. coli/Salmonella* core metabolic network (iSalcoli_core) consisting of a total of 683 ORFs and 1,601 reactions (Table 1), and the reactions remaining were classified based on metabolic subsystem (Figure 3).

### Generation of an enterobacterial core metabolic network

All members of the family of enterobacteria are thought to have diverged from a common ancestor ~300-500 mya [26]. The metabolic network present at that time represents the backbone on which the metabolism of all modern enterobacteria was built. We again assume that genes conserved across all strains represent a conservative estimate of the core genome of the ancestor of modern enterobacterial strains and from these 72 genomes the collection of 756 conserved genes to construct iEntero_core (Figure 2). There are 325 of these genes that have characterized metabolic function in the *E. coli* K-12 MG1655 GEM (iEco1339_MG1655) [4]. A metabolic network for the enterobacterial ancestral core was made by removing reactions from the iEco1339_MG1655 network if one or more of the 72 genomes did not have an orthologous gene to associate to the reaction and if the reaction was not essential. In the case of transport outer membrane porin reactions (n = 247), no single othologous gene was found that spans all 72 enterobacterial genomes, yet four genes (b0241, b0929, b1377, or b2215) have equivalent function for 244 of these reactions in the iEco1339_MG1655 network. For all 72 genomes of enterobacteria examined, one or more of these genes encoding functionally equivalent proteins were found, and led to the retention of these 244 reactions in the enterobacterial core network classified as orphan reactions. Using this approach 1,014 ORFS associated with 937 reactions were removed from iEco1339_MG1655 resulting in an enterobacterial core metabolic network (iEntero_core) consisting of a total of 325 ORFs and 1,191 reactions (Table 1), and the reactions remaining were classified based on metabolic subsystem (Figure 3).

### Assessment and validation of models for carbon source utilization

**Figure 4:**
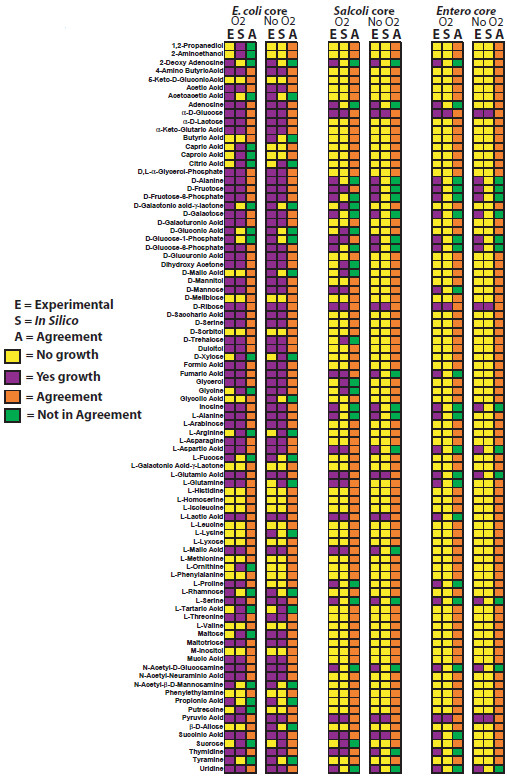
**Experimental and *in silico *carbon source results for *E. coli/Shigella*, *E. coli*/*Shigella*/*Salmonella*, and the enterobacterial ancestral core.**

**Figure 5:**
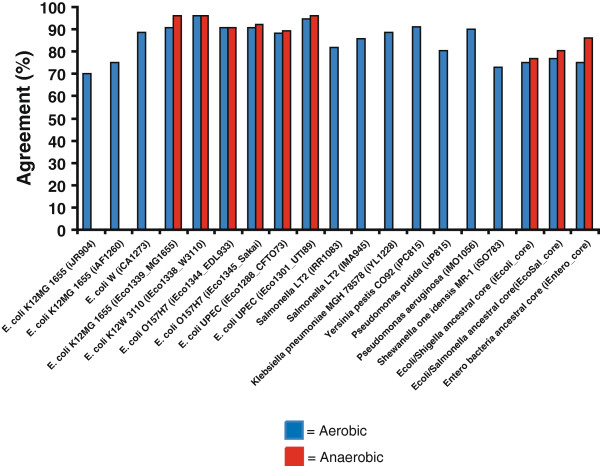
**Comparison of *in silico *carbon source utilization accuracy for *E. coli/Shigella*, *E. coli*/*Shigella*/*Salmonella*, and enterobacterial ancestral core metabolic models in comparison to all other existing GEMs validated with carbon source utilization data.**

To evaluate the accuracy of three ancestral core GEMs, we predicted if these ancestral strains could use 190 different carbon sources under aerobic and anaerobic conditions. These predictions were then compared to experimental growth phenotypes for current strains measured using Biolog phenotypic arrays or published carbon source utilization data for 38 enterobacteria spanning 23 genera listed in Table 2[1,4]. There are numerous strain-specific differences in carbon source utilization in both aerobic (Additional file 1) and anaerobic conditions (Additional file 2). For the experimental growth phenotypes, if any of the current strains could not grow on a carbon source then we assumed the ancestral core model could also not grow on the carbon source. These expected experimental results for the ancestral strains were then compared to FBA predictions of growth for the different ancestral core GEMs using different carbon sources. For those compounds included in the Biolog plates that have transporters in the model, FBA was used to predict if they could be used for growth as sole carbon source. Comparisons were made for all three ancestral metabolic models (Figure 4, Additional file 3). We compared the accuracy of the ancestral models for carbon source predictions to all other microbial GEMs that were validated through a comparison to carbon source utilization data (Figure 5), and determined that the accuracy of carbon source utilization predictions for ancestral metabolic models was similar to the range of accuracy for GEMs of extant bacteria under both aerobic and anaerobic conditions [4,6,10-12,14,16,29-32].

**Table 2:** Sources for experimental carbon source utilization data for modern day enterobacterial strains

**Genus species strain**	**Column number**	**Source or reference**
*Escherichia coli* K-12 MG1655	1	[4] and this study
*Escherichia coli* K-12 W3110	2	[4] and this study
*Escherichia coli* EDL933	3	[4] and this study
*Escherichia coli* Sakai	4	[4] and this study
*Escherichia coli* CFT073	5	[4] and this study
*Escherichia coli* UTI89	6	[4] and this study
*Shigella flexneri* 2457 T	7	This study
*Shigella dysenteriae*	8	[1]
*Shigella boydii*	9	[1]
*Shigella sonnei*	10	[1]
*Salmonella typhimurium* LT2	11	[4] and this study
*Salmonella* Arizonae	12	[1]
*Salmonella* Choleraesuis	13	[1]
*Salmonella* Gallinarum	14	[1]
*Salmonella* Paratyphi	15	[1]
*Salmonella* Typhi	16	[1]
*Citrobacter koseri*	17	[1]
*Enterobacter cloacae*	18	[1]
*Leclercia adecarboxylata*	19	[1]
*Klebsiella pneumoniae*	20	[1]
*Yokenella regensburgei*	21	[1]
*Cronobacter sakazakii*	22	[1]
*Cedecea davisae*	23	[1]
*Erwinia amylovora* ATCC 49946	24	This study
*Pantoea stewartii* DC283	25	This study
*Brenneria salicis*	26	[1]
*Serratia marcescens*	27	[1]
*Ewingella americana*	28	[1]
*Dickeya dadantii* 3937	29	This study
*Pectobacterium atrosepticum* SCRI1043	30	This study
*Yersinia enterocolitica*	31	[1]
*Yersinia pestis*	32	[1]
*Yersinia pseudotuberculosis*	33	[1]
*Edwardsiella tarda*	34	[1]
*Hafnia alvei*	35	[1]
*Photorhabdus luminescens*	36	[1]
*Xenorhabdus nematophila*	37	[1]
*Proteus vulgaris*	38	[1]

### *In silico* predictions for nitrogen, phosphorous, iron, and sulfur utilization predictions

**Figure 6:**
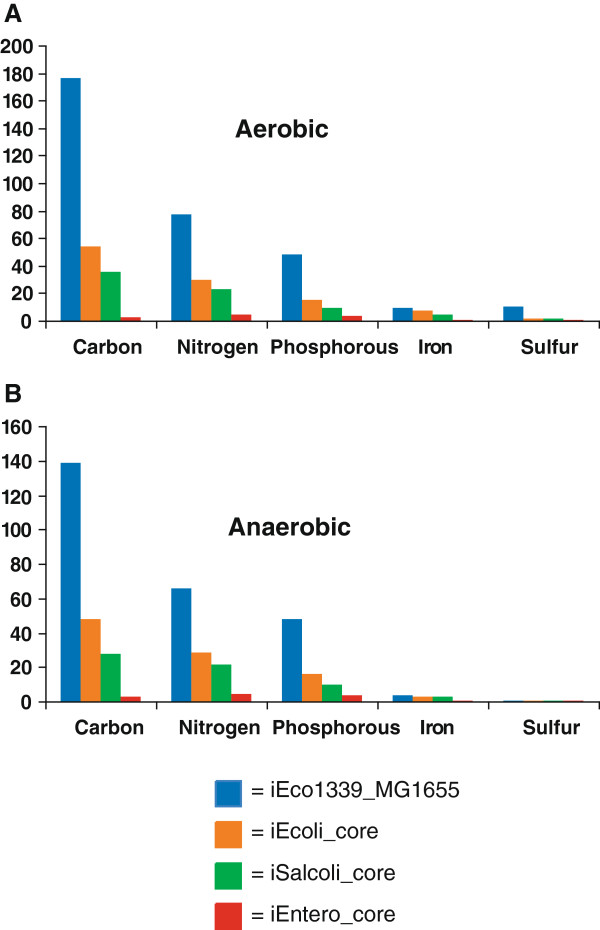
**Carbon, Nitrogen, Phosphorous, Iron, and Sulfur utilization *in silico *predictions for *E. coli/Shigella*, *E. coli*/*Shigella*/*Salmonella*, and the enterobacterial ancestral core metabolic models in (A) aerobic and (B) anaerobic conditions.**

Once all three ancestral core models were validated through comparison to experimental data for determining accuracy of *in silico* carbon source utilization using FBA, predictions were generated for utilization of sole nitrogen, phosphorous, iron, and sulfur compounds. As the number of genomes used for the generation of the three ancestral core models increased, the number of metabolites decreased that were predicted as useable nitrogen, phosphorous, iron, or sulfur source under aerobic (Figure 6A) or anaerobic conditions (Figure 6B).

### Analysis of gene essentiality

**Figure 7:**
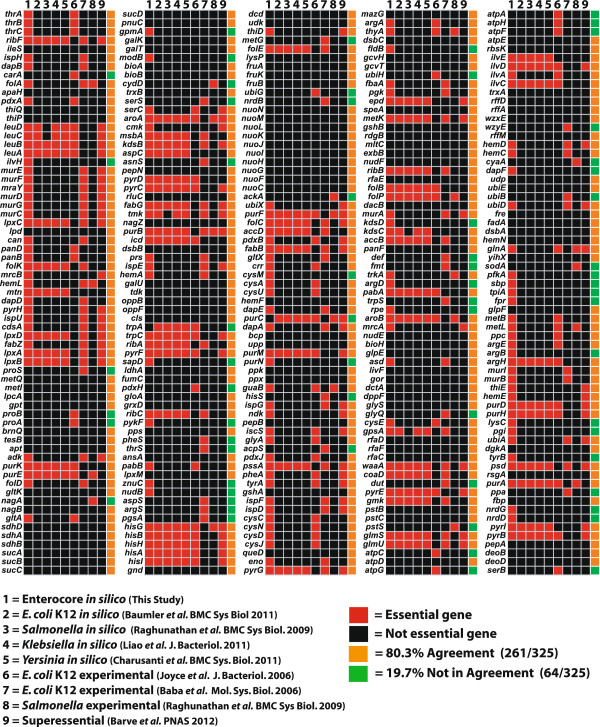
**Comparison of the enterobacterial ancestral core metabolic model gene content to predicted essential genes from *in silico *predictions and to experimentally determined essential genes.**

To further explore the metabolic similarities between all enterobacteria, we determined reaction essentiality predictions of the enterobacterial core metabolic network (iEntero_core) for conditions simulating aerobic and anaerobic growth in glucose containing minimal media (Table 3). Of the 325 genes included in the enterobacterial ancestral core model, we compared the 169 genes predicted *in silico* as essential to orthologous genes in other enterobacteria strains for which GEMs have been constructed [4,11,12,14], to experimentally determined essential genes [14,33,34], and to “superessential” gene predictions (required in all metabolic networks analyzed [35]) (Figure 7). 39% of genes predicted as essential (66/169) using the enterobacterial ancestral core metabolic network were also predicted as essential *in silico* in one or more GEMs generated from genomes of extant enterobacteria. Of the 325 genes contained in the enterobacterial ancestral core metabolic network, 156 genes were predicted as non-essential and 98% (154/156) of these predictions matched non-essential gene predictions from orthologous genes contained in GEMs generated from genomes of extant enterobacteria.

**Table 3:** Subsystem classification for essential reactions predicted for all metabolic models under anaerobic conditions

**Source**	**iEcoli_core**	**iSalcoli_core**	**iEntero_core**
Essential reactions	284	286	326
Essential reactions subsystem classification			
Alternate Carbon Metabolism	2	2	2
Amino Acid Metabolism	82	82	84
Cell Envelope Biosynthesis	45	45	45
Citric Acid Cycle	4	4	5
Cofactor and Prosthetic Group Biosynthesis	66	66	69
Folate Metabolism	2	2	4
Glycerophospholipid Metabolism	10	10	10
Glycolysis	1	1	10
Inorganic Ion Transport and Metabolism	8	9	12
Lipopolysaccharide Biosynthesis / Recycling	11	11	11
Membrane Lipid Metabolism	2	2	4
Murein Biosynthesis	2	2	2
Pentose Phosphate pathway	2	2	3
Purine and Pyrimidine Metabolism	26	26	34
Transport, Inner Membrane	4	5	8
Transport, Outer Membrane	15	15	19
Unassigned	2	2	4

When the predicted 169 genes predicted as essential using the enterobacterial ancestral core metabolic model were compared to experimental data for modern day enterobacterial strains, 39% of essential gene predictions (66/169) were in agreement with the experimentally determined essential genes for *E. coli* and *Salmonella* strains, and out of the 156 non-essential gene predictions generated using the enterobacterial ancestral core metabolic network 74.3% were in agreement with experimentally determined non-essential genes (116/156) (Figure 7). When gene essentiality predictions generated using the enterobacterial ancestral core metabolic network were compared to “superessential” genes, 43.7% (74/169) were in agreement for essential gene predictions and 96.7% were in agreement for non-essential gene predictions (151/156). For each of these comparative gene essentiality data sets, overall predictions using the enterobacterial ancestral core metabolic model were in agreement with gene essentiality predictions from *in silico* enterobacterial GEMs for 68% (221/325) of gene essentiality predictions, 76% (241/325) when compared to experimental data from enterobacterial members, and 70.4% (229/325) when compared to “superessential” gene predictions. In summary, essential gene predictions from the enterobacterial ancestral core were 80.3% in agreement with essentiality data determined previously in one or more published studies from individual enterobacterial members (Figure 7).

## Discussion

This study describes the generation of three computational models designed as a first approximation of the metabolic capacity of ancestral enterobacteria. We selected three nodes in a well resolved phylogenetic tree of enterobacteria with complete genome sequences (Figure 1). The first node represents the common ancestor of *E. coli* and *Shigella* (~10 mya), the second represents the ancestor of *Ecoli/Shigella* and *Salmonella* (~100 mya), and the third represents the ancestor of all enterobacteria (~300-500 mya). Each model includes all identifiable metabolic reactions from a previous GEM for *E. coli* K-12 that are linked to genes conserved among (23, 39 and 72 genomes, respectively) descendants of the phylogenetic node, plus orphan reactions from the *E. coli* K-12 model that must be retained because removal would prevent production of biomass. Thus, these three models are progressively smaller subsets of the *E. coli* K-12 model. The models were created in a step-wise fashion by deleting reactions missing from one taxon at a time selected approximately in order of increasing phylogenetic distance from *E. coli* K-12. Figure 2 shows the impact of each successive addition on the number of genes and reactions retained. The number of orphan reactions is also shown in Figure 2 and increases with divergence time of the ancestral node.

For the model representing the ancestor of all enterobacteria, over 600 of the approximately 1,200 reactions in the model are orphans (Figure 2). There are several possible explanations. These orphans may arise from false-negative ortholog predictions that support removing a reaction that appears to be missing from a taxon, but for which the genes are truly present in the annotated genome. Similarly, orphans could arise from false-negative gene prediction errors. Both these mundane error sources could be corrected manually, but this would require a good deal of effort. A more interesting explanation is that these reactions might be carried out by non-orthologous isofunctional equivalents in the organisms that suggested removing them from the model. This was the case for 244 transport outer membrane porin reactions that were retained in the enterobacterial ancestral core model that did not have a single ortholog conserved across all 72 genomes, yet each of these genomes contained one or more functionally equivalent genes encoding isozymes for these reactions. Further examination of these cases could advance understanding of the rates and patterns of non-orthologous displacement in the evolution of metabolic processes. It is also likely that we will see a reduction of orphan reactions in the next generation of ancestral models that makes use of parsimony or maximum likelihood based ancestral state prediction to determine which genes are present at each internal node, because this approach will be more tolerant of annotation or orthology errors affecting a subset of taxa. It will also expand the models to include reactions that were legitimately lost in individual taxa and lineages allowing us to further probe the evolutionary dynamics of metabolic networks and perhaps discover system-scale emergent phenotypes linked with losses of particular reactions.

Here, we focus on these models representing core absolutely conserved reactions to examine a conservative estimate of the metabolic capacity of each ancestral lineage. We compared the reactions retained in each ancestral model to the total set of reactions in the mature *E. coli* K-12 model for each reaction subsystem classification category (Figure 3). Some subsystems are particularly highly conserved. *Murein biosynthesis* and *transport outer membrane porin* reactions are almost entirely conserved across all phylogenetic depths assayed. Other categories, like *alternate carbon metabolism*, *transport inner membrane* and *glycerophospholipid metabolism* are highly variable across the models. For example, 119 reactions involved with *alternate carbon metabolism* were deleted from the *E. coli* K-12 model in order to create the *E. coli* ancestral core model, 9 additional deletions were required for the *E. coli/Salmonella* ancestral core model, and 27 more were required for the enterobacteria ancestral core model. This pattern suggests that much of the variation in this biological process lies at the species level. In contrast, for the *glycerophospholipid metabolism* subsystem, the *E. coli/Shigella* core and *E. coli/Salmonella* core models are identical, but 116 reaction deletions were required to create the enterobacteria core model. This tells us that this metabolic process was largely intact by the time *E. coli* and *Salmonella* diverged, but these metabolic capabilities were likely acquired since the ancestor of all enterobacteria. Future modeling of additional nodes with incorporation of ancestral state reconstruction will further illuminate the timing of these metabolic innovations. This in turn, can be used to simulate metabolic evolution in the forward direction, adding capabilities in step-wise evolutionary order. With each addition, it will be possible to compete each “evolved” strain against its ancestor *in silico* altering the environmental conditions in the simulation to examine the relative predicted biomass (i.e. fitness).

Since the biomass of ancestral cells is impossible to determine empirically, we used the *E. coli* Biomass equation from iAF1260 [6] for all ancestral core metabolic models in this study. The iAF1260 biomass equation is the most extensive biomass equation of all enterobacterial GEMs containing 92 metabolites including many trace metals and elements such as iron and sulfur that are known to be essential for supporting microbial life. Since unique biomass equations exist for three other strains of enterobacteria with GEMs [11,12,14], we compared the metabolites present in all strain-specific enterobacterial GEMs and determined that there are 34 present in all four biomass equations, 8 in three of the four, 18 in two of the four, and that there are metabolites specific to each strain-specific biomass equation for *E. coli (23)*, *Salmonella (8)*, *Klebsiella (12)*, and *Yersinia (6)*. As more enterobacterial members have GEMs constructed with strain-specific biomass equations, a future direction may be to generate an average biomass composition from all modern-day GEMs of enterobacteria to use in the enterobacterial ancestral core metabolic model, or to use parsimony-based ancestral state reconstruction to estimate node-specific ancestral biomass composition. Future studies are warranted to investigate whether and how these alternative approaches impact insights gained through simulations.

We used the models to predict whether each ancestral “strain” would grow on a large number of carbon, nitrogen, phosphorous, iron, and sulfur sources under aerobic and anaerobic conditions. We compared these predictions to previously published experimental growth data for 38 extant enterobacteria representing the 23 genera used in the phylogenetic analysis (Figure 1) (several of which were not represented among the genomes used to construct our models) to investigate the accuracy of the models (Figure 4). For each node corresponding to one of our models, we first compiled published experimental data recording whether each nutrient could serve as a sole source for growth in all reported wild-type descendants of the node. If even a single report indicated that a strain was unable to utilize the nutrient as a sole source, we recorded it as a negative. For the *E. coli/Shigella*, *Ecoli/Shigella/Salmonella*, and the enterobacteria core model reconstructions, the total number of experimentally reported carbon sources utilized by all relevant strains in anaerobic conditions were 81, 65, and 33, and in anaerobic conditions were 77, 46, and 16, respectively (Figure 4). Previously it was appreciated that all free-living enterobacteria could utilize glucose as a sole carbon source [1]. Our compilation of experimental data identified 15 additional carbon sources that are utilized by all free-living enterobacteria (alpha-D-Glucose, D, L-Malic Acid, D-Alanine,D-Fructose,D-Fructose-6-Phosphate, D-Galactose, D-Glucose-1-Phosphate, D-Glucose-6-Phosphate, D-Ribose, Inosine, L-Aspartic Acid, L-Glutamic Acid, L-Serine, N-Acetyl-D-Glucosamine, Pyruvic Acid, and Uridine). Catabolism of these substrates has been conserved in all free-living enterobacteria as they diverged over ~300-500 million years.

Once experimental data was summarized, we sought to determine how well the ancestral core metabolic models accurately predicted carbon source utilization phenotypes. In order to compare experimental data (Additional files 1,2, and 3) to *in silico* predictions, experimental growth data was summarized for the members contained at each major branching point for *E. coli/Shigella* (strains 1–10 in Table 2), *Ecoli/Shigella/Salmonella* (strains 1–16 in Table 2), and the enterobacteria (strains 1–38 in Table 2). Growth or no-growth data was then determined, and if one member had a no growth phenotype, the consensus of experimental data to compare with ancestral core metabolic models was no growth. For each experimental growth prediction, all of the strains with experimental data had to be positive for growth or not determined for a positive growth prediction to compare with *in silico* ancestral core predictions. Of the 190 carbon sources with experimental data, 87 of these are present with exchange and transport reactions in the metabolic ancestral core models and thus provide an opportunity for a comparison between experimental and *in silico* carbon source utilization predictions. Based on these 87 carbon sources, the accuracy of the three models, iEcoli_core, iSalcoli_core, and iEntero_core, for aerobic carbon source utilization predictions were 75, 77, and 75%, and in anaerobic conditions were 76, 80.5, and 86%, respectively (Figure 4). When compared to all existing microbial GEMs that used experimental carbon source utilization data to validate *in silico* metabolic model predictions, the accuracy observed for the three ancestral metabolic models for aerobic (75.3% ± 1.5) or anaerobic (81.5% ± 4) conditions is within the range of accuracy for all 16 GEMs (86% ± 7.5) that have been published (Figure 5). This supports the use of more general ancestral models in cases where a genome sequence, or specific model, or both are lacking. These core models are also an excellent starting point for generation of additional strain-specific models for other enterobacteria.

Having validated the ancestral core models using a subset of experimentally determined carbon source utilization data, we examined the predictions of each model for a wider variety of carbon, nitrogen, phosphorous, iron, and sulfur source utilization phenotypes (Figure 6). There were three carbon sources (glucose, ribose, and pyruvic acid), five nitrogen sources (D-alanyl-D-alanine, L-asparagine, L-aspartate, glycine, and ammonia), four phosphorous sources (N-Acetyl-D-galactosamine 1-phosphate, N-acetyl-D-glucosamine 1-phosphate, D-glucuronate, and phosphate), one iron source (ferric iron Fe^3+^), and one sulfur source (sulfate SO_4_) predicted to support growth of the ancestral core of enterobacteria (Figure 6). These results provide new insight about the metabolic capability that has been retained in almost all free-living enterobacteria over ~300-500 mya of divergence.

Finally, we examined which genes/reactions are predicted as essential based on the enterobacteria ancestral core metabolic model. We conducted *in silico* gene essentiality analysis of all 325 genes contained in the iEntero_core model, and identified 169 genes/reactions that were predicted to be essential for growth. We compared these predictions to available *in silico* and experimental essential gene data for extant strains of enterobacteria. When genes predicted as essential for iEntero_core were compared to *in silico* predictions of GEMs for *E. coli* K12 [4], *Salmonella*[14], *Klebsiella*[12], and *Yersinia*[11], 63 enterobacterial ancestral core metabolic model essential gene predictions matched the essential genes predicted for all four enterobacterial strain-specific GEMs, and five predicted essential genes matched essential gene predictions for 3 out of 4 strain-specific GEMs (Figure 7). When genes predicted as essential for iEntero_core were compared to experimental data for *E. coli* K12 [33,34] or *Salmonella*[14], 128 iEntero_core essential genes matched at least one of the experimental essential gene predictions for *E. coli* K12, and 15 matched experimental essential gene predictions for *Salmonella*. In addition “superessential” genes identified for reactions essential to almost all prokaryotic organisms [35] were compared to enterobacterial core predictions, and 74 were found to match genes predicted as essential and 116 predicted as nonessential. Overall, gene essentiality predictions using the iEntero_core ancestral model were in 80.3% (n = 325) agreement with at least one other data set from previously published data (Figure 7). This analysis also pinpoints which genes have been identified or predicted to be essential across nearly all studies, and these may represent the best targets for the generation of antibiotics or other control strategies for infectious diseases associated with enterobacteria.

## Conclusions

The work presented here are the most advanced and comprehensive quantitative ancestral metabolic models to date to investigate metabolism through genomic comparison of extant descendants, and has provided insight into aspects of metabolism of ancient microbes. We showed evidence that different subsystems of the metabolic network evolved according to different rates and patterns. This includes subsystems that vary extensively within species after remaining relatively stable for much longer evolutionary times, as well as subsystems whose composition has been retained in all extant strains. This work demonstrated a new approach for validation of carbon source utilization of ancestral models, yielding accuracies of >75%, for aerobic and anaerobic conditions for the ancestral core models for *Ecoli/Shigella*, *E. coli*/*Shigella*/*Salmonella*, and enterobacteria. Importantly, the ancestral core models showed comparable accuracy to organism-specific models suggesting they will be useful starting points for modeling lesser-characterized enterobacteria. Essential gene predictions for the enterobacterial ancestral core were compared to extensive experimental data and revealed the most promising new targets for future development of control strategies such as new broad-spectrum antibiotics to treat disease caused by enterobacteria. These insights support the use of this “paleo systems biology” approach to study ancient metabolism and metabolic network evolution through reconstruction of models of ancestral lineages that are otherwise inaccessible for experimentation.

## Materials and methods

### Bacterial strains and growth conditions

Six *E. coli* strains, one *Salmonella* strain, one *Shigella* strain, one *Erwinia* strain, one *Pantoea* strain, one *Dickeya* strain, and one *Pectobacterium* strain were used in this study (Table 4). Frozen cultures were streaked onto Luria Bertani (LB) agar plates and grown overnight at 37°C for *E. coli*, *Salmonella*, and *Shigella* strains, and at 28°C for the *Erwinia*, *Pantoea*, *Dickeya*, and *Pectobacterium* strains. For carbon plate utilization assays, isolated colonies were used to inoculate BUG Sheep Blood Agar plates (Biolog, Hayward, CA) and incubated at 37°C or 28°C overnight aerobically or anaerobically in sealed Whirl-Pak® Long-Term Sample Retention Bags (Nasco, Fort Atkinson, Wisconsin) saturated with an anaerobic gas mixture (95% N_2_ and 5% CO_2_) as described [4]. Cells were collected and used to inoculate Biolog PM1 and PM2 plates following the manufacturers recommendations with a minor modification of adding a top layer of mineral oil to each well for anaerobic culture conditions as described [4], and Biolog plates were monitored for up to 48 h for the *E. coli*, *Salmonella*, and *Shigella* strains, and up to 72 h for the *Erwinia*, *Pantoea*, *Dickeya*, and *Pectobacterium* strains.

**Table 4:** List of bacterial strains used in this study

**Strain**	**Genotype**	**Source or reference**
*E. coli* K-12 MG1655	Wild type	Dr. Patricia J. Kiley, University of Wisconsin-Madison [45]
*E. coli* K-12 W3110	Wild type	ATCC 39936
*E. coli* O157:H7 EDL933	Wild type	Dr. Charles W. Kaspar, University of Wisconsin-Madison [46]
*E. coli* O157:H7 RIMD/Sakai	Wild type	ATCC BAA-460 [47]
*E. coli* CFT073	Wild type	Dr. Rodney A. Welch, University of Wisconsin-Madison [48]
*E. coli* UTI89	Wild type	Dr. Scott J. Hultgren, Washington University, St. Louis [49]
*Shigella flexneri* 2457 T	Wild type	Dr. Nicole T. Perna, University of Wisconsin-Madison [50]
*Salmonella enteric* serovar *Typhimurium* LT2	Wild type	Dr. Diana M. Downs, University of Wisconsin-Madison [51]
*Erwinia amylovora* ATCC 49946	Wild type	Dr. Nicole T. Perna, University of Wisconsin-Madison [52]
*Pantoea stewartii* DC283	Wild type	Dr. Nicole T. Perna, University of Wisconsin-Madison
*Dickeya dadantii* 3937	Wild Type	Dr. Nicole T. Perna, University of Wisconsin-Madison [53]
*Pectobacterium atrosepticum* SCRI1043	Wild type	Dr. Nicole T. Perna, University of Wisconsin-Madison

### Genome-wide phylogenetic reconstruction

Genomes used in this study for phylogenetic reconstruction, their sources, dates of isolation, and hosts are listed in Additional file 4. Out of the total 44 genera and 176 named species for the family of *Enterobacteriaceae*[1], there are over 147 complete or nearly complete genomes from 23 genera currently available, according to NCBI microbial genome project and ASAP databases [36]. For all strains with available genomes, one strain from each genus was selected for phylogenetic analysis from those contained in the ASAP database *Vibrio cholerae* and *Pseudomonas syringae* were designated as outgroup taxa, because they are members of phylogenetically closely related families from the order of gamma proteobacteria. Genome-wide orthologous genes among selected genomes were retrieved in two steps. We first generated all-against-all BLASTP reciprocal (best or nearly best) matches for all investigated sequences, using an E-value ≤ 0.000001 cutoff. We then use a threshold based on a metric that is defined as the minimal number of pair-wise comparisons consistent across a putative orthologous sequence cluster, in order to preserve a genome-wide dataset with maximal phylogenetic informativeness. Alignment of each retrieved orthologous data among all strains under investigation was performed in AMAP (Protein multiple alignment by sequence annealing) [37] with 0.5 as the gap factor. Amino acid sequence alignments were concatenated to form a single composite alignment. We further employed both neighbor joining (NJ) and the maximum likelihood estimation (MLE) for phylogenetic reconstruction. NJ trees were calculated in PAUP* 4.0b10 [38,39] and maximum likelihood trees were constructed in PhyML [40] for both individual genes and the composite data sets. BioNJ tree [41] is used as starting tree topology in PhyML, and optimized tree topology and optimized branch lengths and rate parameters are also used. WAG (Whelan And Goldman) is employed as the amino acid substitution model [42] and gamma distribution parameter and proportion of invariable sites are estimated using four substitution rate categories are used. The 50% majority-rule consensus trees were calculated using 1000 bootstrap pseudo-replicates with sampling limited to non-excluded, parsimony-informative characters.

### Generation of ancestral metabolic networks

Draft and complete enterobacterial genomes in the ASAP database have been continually updated using new publicly accessible genomes since the database’s inception [36]. Orthologs in the ASAP database are derived from multiple criteria, including pairwise reciprocal BLASTP searches filtered with comparison-specific thresholds for percent identity restricted to hits encompassing > 60% of both aligned proteins, followed by manual curation based on local and larger-scale conservation of genome context as well as expert review of alignments, and comparison to large-scale OrthoMCL analyses [36]. There are more than 300 genomes of enterobacteria in the ASAP database, of which 72 genomes (spanning 16 genera) were chosen that all have gene orthology predictions determined in comparison to *E. coli* K12 MG1655 (Table 5). Using the phylogenetic tree as a guide, we generated a table of genes (rows) contained in the iEco1339_MG1655 metabolic model [4] with the 72 genomes (columns) and column entries correspond to orthologous ASAP gene identifiers, with blank entries representing cases in which no orthologous gene exists (Additional file 5). We then chose three phylogenetic branching points to identify genomes representing the conserved ancestral core for *E. coli/Shigella* (genomes #1-23), *Ecoli/Shigella/Salmonella* (genomes #1-39), and the enterobacterial core (genomes #1-72). The GEMs for these ancestral core models were made by removing orthologous ORFs and their associated reactions from the iEco1339_MG1655 GEM if one or more of the enterobacteria genomes leading up to the phylogenetic branching point did not have a gene assigned. If removing a reaction prevented biomass production for anaerobic growth on glucose (predicted using FBA) then the reaction was added back to the metabolic reconstruction without a gene associated with it. Gene-to-protein-to-reaction associations representing the metabolic models of the *E. coli/Shigella* (iEcoli_core)*,E.coli/Shigella/Salmonella* (iSalcoli_core)*,* and the *Enterobacterial* (iEntero_core) ancestral core are provided (Additional file 6). Ancestral core metabolic models were converted to SBML file format and are provided for iEcoli_core (Additional file 7), iSalcoli_core (Additional file 8), and iEntero_core (Additional file 9).

**Table 5:** Genomes used to construct ancestral core metabolic networks

**Strain**	**ORFs**	**Genome number**
*Escherichia coli* K-12 MG1655	4,141	1
*Escherichia coli* EDL933	5,196	2
*Escherichia coli* 53638	5,172	3
*Escherichia coli* CFTO73	4,889	4
*Escherichia coli* E2348/69	4,652	5
*Escherichia coli* EC4115	5,467	6
*Escherichia coli* UTI89	4,944	7
*Escherichia coli* E24377A	4,953	8
*Escherichia coli* Sakai	5,253	9
*Escherichia coli* SE11	4,973	10
*Escherichia coli* APEC O1	5,045	11
*Escherichia coli* SMS-3-5	4,906	12
*Escherichia coli* 536	4,599	13
*Escherichia coli* HS	4,393	14
*Escherichia coli* ATCC 8739	4,236	15
*Escherichia coli* K-12 W3110	4,171	16
*Shigella boydii* 227	4,578	17
*Shigella boydii* BS512	4,578	18
*Shigella dysenteriae* 197	4,460	19
*Shigella flexneri* 2457 T	4,527	20
*Shigella flexneri* 301	4,460	21
*Shigella flexneri* 8401	4,135	22
*Shigella sonnei* 046	4,456	23
*Salmonella* Agona SL483	4,613	24
*Salmonella* Arizonae CDC 346-86	4.505	25
*Salmonella* Choleraesuis SC-B67	4,663	26
*Salmonella* Dublin CT_02021853	4,619	27
*Salmonella* Enteritidis P125109	4,204	28
*Salmonella* Gallinarum 287/91	3,963	29
*Salmonella* Heidelberg SL476	4,779	30
*Salmonella* Newport SL254	4,807	31
*Salmonella* Paratyphi A AKU_12601	4,286	32
*Salmonella* Paratyphi A ATCC 9150	4,095	33
*Salmonella* Paratyphi B SPB7	5,590	34
*Salmonella* Schwarzengrund CVM19633	4,628	35
*Salmonella* Typhi CT18	4,696	36
*Salmonella* Typhi Ty2	4,323	37
*Salmonella Typhimurium* 14028S	5,474	38
*Salmonella Typhimurium* LT2	4,525	39
*Leclercia adecarboxylata* ATCC 23216	4,732	40
*Klebsiella pneumoniae* MGH 78578	5,185	41
*Yokenella regensburgei* ATCC 49455	4,657	42
*Cedecea davisae* ATCC 33431	4,590	43
*Erwinia amylovora* ATCC 49946	3,616	44
*Erwinia tasmaniensis* Et1/99	3,623	45
*Pantoea stewartii* DC283	4,964	46
*Serratia marcescens* subsp. *marcescens* ATCC 13880	4,892	47
*Ewingella americana* ATCC 33852	4,444	48
*Dickeya dadantii* 3937	4,494	49
*Dickeya sp.i* Ech586	4,215	50
*Dickeya* sp. 703	3,970	51
*Dickeya sp* Ech1591	4,162	52
*Pectobacterium atrosepticum* SCRI1043	4,466	53
*Pectobacterium brasiliensis* 1692	5,127	54
*Pectobacterium carotovorum* PC1	4,245	55
*Pectobacterium carotovorum* WPP14	4,818	56
*Pectobacterium wasabiae* WPP163	4,507	57
*Yersinia enterocolitica* 8081	4,054	58
*Yersinia pestis* 91001	4,190	59
*Yersinia pestis* Angola	4,044	60
*Yersinia pestis* Antiqua	4,357	61
*Yersinia pestis* CA88-4125	4,115	62
*Yersinia pestis* CO92	3,986	63
*Yersinia pestis* KIM	4,321	64
*Yersinia pestis* Nepal516	4,085	65
*Yersinia pestis* Pestoides F	4,063	66
*Yersinia pseudotuberculosis* IP31758	4,324	67
*Yersinia pseudotuberculosis* IP32953	4,058	68
*Yersinia pseudotuberculosis* PB1/+ 1	4,235	69
*Yersinia pseudotuberculosis* YPIII	4,190	70
*Hafnia alvei* ATCC 13337	4,509	71
*Photorhabdus luminescens* TTO1	4,684	72

### Flux balance analysis

Fluxes through metabolic network reactions can be predicted using flux balance analysis (FBA) [43]. In FBA, fluxes are constrained by steady-state mass balances, enzyme capacities and reaction directionality. These constraints yield a solution space of possible flux values, and FBA uses an objective function to identify flux distributions that maximize (or minimize) the physiologically relevant predicted solution. Cellular growth rate (or biomass production) is often used as an objective function for FBA [44], and was used for FBA analyses performed in this study in addition to an objective function for respiration which has been shown to improve comparisons to Biolog carbon source experimental data [4]. The same biomass equation, GAM and NGAM values, and PO ratio were used for all developed models, and were the same as that in iAF1260 [6]. Using FBA, *in silico* predictions of carbon, nitrogen, phosphorous, iron, and sulfur source utilization were compared to experimentally determined values for 38 enterobacterial strains spanning 23 genera for both aerobic and anaerobic conditions. For carbon, nitrogen, phosphorous, iron, and sulfur source utilization and gene deletion simulations, a maximum uptake rate of 10 mmol per gram of dry weight per hour (mmol/gDW cell/h) was used. FBA was also used to predict essential reactions by constraining reactions to have zero flux and maximizing growth rate. If the resulting maximum predicted growth rate (using FBA) was zero then the reaction and the associated genes were considered to be essential. Reaction deletion simulations were evaluated under both aerobic and anaerobic conditions.

## Competing interest

The authors declare that they have no conflict of interest.

## Authors’ contribution

NP conceptualized and DB designed the study. BM conducted all phylogenetic analysis, and NP and BM analyzed the results. DB constructed all three ancestral core metabolic network reconstructions and performed all *in silico* analyses. DB obtained all of the experimental data. DB and JR analyzed and interpreted the data and performed the statistical analysis. All authors helped draft and edit the final manuscript. DB generated all SBML model files. All authors approve the content of this manuscript. All authors read and approved the final manuscript.

## Supplementary Material

Additional file 1 Aerobic experimental carbon source utilization for 38 enterobacterial strains. Click here for file

Additional file 2 Anaerobic experimental carbon source utilization for 38 enterobacterial strains. Click here for file

Additional file 3 **Dataset 1.** A list of experimental carbon source utilization data of enterobacteria. Click here for file

Additional file 4: Table S1 List of genomes used for the phylogenetic analysis of the enterobacteria. Click here for file

Additional file 5 **Dataset 2.** A list of orthologous genes from 72 genomes of enterobacteria to those from *E. coli* K-12 MG1655, and to genes contained in the metabolic models of the *E. coli/Shigella* (iEcoli_core)*,E.coli/Shigella/Salmonella* (iSalcoli_core) *,* and the enterobacterial (iEntero_core) ancestral core. Click here for file

Additional file 6 **Dataset 3.** Gene-to-protein-to-reaction associations representing the metabolic models of the *E. coli/Shigella* (iEcoli_core)*, E.coli/Shigella/Salmonella* (iSalcoli_core)*,* and the enterobacterial (iEntero_core) ancestral core. Click here for file

Additional file 7 **Computational Model 1.** SBML format of iEcoli_core for distribution and use in other modeling environments. Click here for file

Additional file 8 **Computational Model 2.** SBML format of iSalcoli_core for distribution and use in other modeling environments. Click here for file

Additional file 9 **Computational Model 3.** SBML format of iEntero_core for distribution and use in other modeling environments. Click here for file

## References

[B1] Brenner DJ Brenner NRK DJ, Staley JT FIJJ Family I. Enterobacteriaceae Bergey‘s Manual of Systematic Bacteriology 2005 New York: Springer 587 850

[B2] Feist AM Herrgard MJ Thiele I Reed JL Palsson BO Reconstruction of biochemical networks in microorganisms Nat Rev Microbiol 2009 7 129 143 19116616 10.1038/nrmicro1949 PMC3119670

[B3] Oberhardt MA Palsson BO Papin JA Applications of genome-scale metabolic reconstructions Mol Syst Biol 2009 5 320 19888215 10.1038/msb.2009.77 PMC2795471

[B4] Baumler DJ Peplinski RG Reed JL Glasner JD Perna NT The evolution of metabolic networks of E. coli BMC Syst Biol 2011 5 182 10.1186/1752-0509-5-182 22044664 PMC3229490

[B5] Covert MW Knight EM Reed JL Herrgard MJ Palsson BO Integrating high-throughput and computational data elucidates bacterial networks Nature 2004 429 92 96 10.1038/nature02456 15129285

[B6] Feist AM Henry CS Reed JL Krummenacker M Joyce AR Karp PD Broadbelt LJ Hatzimanikatis V Palsson BO A genome-scale metabolic reconstruction for Escherichia coli K-12 MG1655 that accounts for 1260 ORFs and thermodynamic information Mol Syst Biol 2007 3 121 17593909 10.1038/msb4100155 PMC1911197

[B7] Orth JD Conrad TM Na J Lerman JA Nam H Feist AM Palsson BO A comprehensive genome-scale reconstruction of Escherichia coli metabolism–2011 Mol Syst Biol 2011 7 535 21988831 10.1038/msb.2011.65 PMC3261703

[B8] Reed JL Vo TD Schilling CH Palsson BO An expanded genome-scale model of Escherichia coli K-12 (iJR904 GEM/GPR) Genome Biol 2003 4 R54 10.1186/gb-2003-4-9-r54 12952533 PMC193654

[B9] Thiele I Jamshidi N Fleming RM Palsson BO Genome-scale reconstruction of Escherichia coli‘s transcriptional and translational machinery: a knowledge base, its mathematical formulation, and its functional characterization PLoS Comput Biol 2009 5 e1000312 10.1371/journal.pcbi.1000312 19282977 PMC2648898

[B10] Archer CT Kim JF Jeong H Park JH Vickers CE Lee SY Nielsen LK The genome sequence of E. coli W (ATCC 9637): comparative genome analysis and an improved genome-scale reconstruction of E. coli BMC Genomics 2011 12 9 10.1186/1471-2164-12-9 21208457 PMC3032704

[B11] Charusanti P Chauhan S McAteer K Lerman JA Hyduke DR Motin VL Ansong C Adkins JN Palsson BO An experimentally-supported genome-scale metabolic network reconstruction for Yersinia pestis CO92 BMC Syst Biol 2011 5 163 10.1186/1752-0509-5-163 21995956 PMC3220653

[B12] Liao YC Huang TW Chen FC Charusanti P Hong JS Chang HY Tsai SF Palsson BO Hsiung CA An experimentally validated genome-scale metabolic reconstruction of Klebsiella pneumoniae MGH 78578, iYL1228 J Bacteriol 2011 193 1710 1717 10.1128/JB.01218-10 21296962 PMC3067640

[B13] Navid A Almaas E Genome-scale reconstruction of the metabolic network in Yersinia pestis, strain 91001 Mol Biosyst 2009 5 368 375 10.1039/b818710j 19396373

[B14] Raghunathan A Reed J Shin S Palsson B Daefler S Constraint-based analysis of metabolic capacity of Salmonella typhimurium during host-pathogen interaction BMC Syst Biol 2009 3 38 10.1186/1752-0509-3-38 19356237 PMC2678070

[B15] Thomas GH Zucker J Macdonald SJ Sorokin A Goryanin I Douglas AE A fragile metabolic network adapted for cooperation in the symbiotic bacterium Buchnera aphidicola BMC Syst Biol 2009 3 24 10.1186/1752-0509-3-24 19232131 PMC2649895

[B16] AbuOun M Suthers PF Jones GI Carter BR Saunders MP Maranas CD Woodward MJ Anjum MF Genome scale reconstruction of a Salmonella metabolic model: comparison of similarity and differences with a commensal Escherichia coli strain J Biol Chem 2009 284 29480 29488 10.1074/jbc.M109.005868 19690172 PMC2785581

[B17] Alper H Jin YS Moxley JF Stephanopoulos G Identifying gene targets for the metabolic engineering of lycopene biosynthesis in Escherichia coli Metab Eng 2005 7 155 164 10.1016/j.ymben.2004.12.003 15885614

[B18] Fong SS Burgard AP Herring CD Knight EM Blattner FR Maranas CD Palsson BO In silico design and adaptive evolution of Escherichia coli for production of lactic acid Biotechnol Bioeng 2005 91 643 648 10.1002/bit.20542 15962337

[B19] Lee SJ Lee DY Kim TY Kim BH Lee J Lee SY Metabolic engineering of Escherichia coli for enhanced production of succinic acid, based on genome comparison and in silico gene knockout simulation Appl Environ Microbiol 2005 71 7880 7887 10.1128/AEM.71.12.7880-7887.2005 16332763 PMC1317394

[B20] Lee KH Park JH Kim TY Kim HU Lee SY Systems metabolic engineering of Escherichia coli for L-threonine production Mol Syst Biol 2007 3 149 18059444 10.1038/msb4100196 PMC2174629

[B21] Park JH Lee KH Kim TY Lee SY Metabolic engineering of Escherichia coli for the production of L-valine based on transcriptome analysis and in silico gene knockout simulation Proc Natl Acad Sci U S A 2007 104 7797 7802 10.1073/pnas.0702609104 17463081 PMC1857225

[B22] Reed JL Patel TR Chen KH Joyce AR Applebee MK Herring CD Bui OT Knight EM Fong SS Palsson BO Systems approach to refining genome annotation Proc Natl Acad Sci U S A 2006 103 17480 17484 10.1073/pnas.0603364103 17088549 PMC1859954

[B23] Feist AM Palsson BO The growing scope of applications of genome-scale metabolic reconstructions using Escherichia coli Nat Biotechnol 2008 26 659 667 10.1038/nbt1401 18536691 PMC3108568

[B24] Pal C Papp B Lercher MJ Csermely P Oliver SG Hurst LD Chance and necessity in the evolution of minimal metabolic networks Nature 2006 440 667 670 10.1038/nature04568 16572170

[B25] Yizhak K Tuller T Papp B Ruppin E Metabolic modeling of endosymbiont genome reduction on a temporal scale Mol Syst Biol 2011 7 479 21451589 10.1038/msb.2011.11 PMC3094061

[B26] Deng W Burland V Plunkett G 3rd Boutin A Mayhew GF Liss P Perna NT Rose DJ Mau B Zhou S Schwartz DC Fetherston JD Lindler LE Brubaker RR Plano GV Straley SC McDonough KA Nilles ML Matson JS Blattner FR Perry RD Genome sequence of Yersinia pestis KIM J Bacteriol 2002 184 4601 4611 10.1128/JB.184.16.4601-4611.2002 12142430 PMC135232

[B27] Reid SD Herbelin CJ Bumbaugh AC Selander RK Whittam TS Parallel evolution of virulence in pathogenic Escherichia coli Nature 2000 406 64 67 10.1038/35017546 10894541

[B28] Touchon M Hoede C Tenaillon O Barbe V Baeriswyl S Bidet P Bingen E Bonacorsi S Bouchier C Bouvet O Calteau A Chiapello H Clermont O Cruveiller S Danchin A Diard M Dossat C Karoui ME Frapy E Garry L Ghigo JM Gilles AM Johnson J Le Bouguénec C Lescat M Mangenot S Martinez-Jéhanne V Matic I Nassif X Oztas S Petit MA Pichon C Rouy Z Ruf CS Schneider D Tourret J Vacherie B Vallenet D Médigue C Rocha EP Denamur E Organised genome dynamics in the *Escherichia coli* species results in highly diverse adaptive paths PLoS Genet 2009 5 1 e1000344 10.1371/journal.pgen.1000344 19165319 PMC2617782

[B29] Oberhardt MA Puchalka J Fryer KE Santos VA M d Papin JA Genome-scale metabolic network analysis of the opportunistic pathogen Pseudomonas aeruginosa PAO1 J Bacteriol 2008 190 2790 2803 10.1128/JB.01583-07 18192387 PMC2293256

[B30] Pinchuk GE Hill EA Geydebrekht OV De Ingeniis J Zhang X Osterman A Scott JH Reed SB Romine MF Konopka AE Beliaev AS Fredrickson JK Reed JL Constraint-based model of Shewanella oneidensis MR-1 metabolism: a tool for data analysis and hypothesis generation PLoS Comput Biol 2010 6 e1000822 10.1371/journal.pcbi.1000822 20589080 PMC2891590

[B31] Reed JL Shrinking the Metabolic Solution Space Using Experimental Datasets PLoS Comput Biol 2012 8 8 e1002662 10.1371/journal.pcbi.1002662 22956899 PMC3431291

[B32] Puchalka J Oberhardt MA Godinho M Bielecka A Regenhardt D Timmis KN Papin JA Santos VA M d Genome-scale reconstruction and analysis of the Pseudomonas putida KT2440 metabolic network facilitates applications in biotechnology PLoS Comput Biol 2008 4 e1000210 10.1371/journal.pcbi.1000210 18974823 PMC2563689

[B33] Baba T Ara T Hasegawa M Takai Y Okumura Y Baba M Datsenko KA Tomita M Wanner BL Mori H Construction of Escherichia coli K-12 in-frame, single-gene knockout mutants: the Keio collection Mol Syst Biol 2006 2 2006 0008 16738554 10.1038/msb4100050 PMC1681482

[B34] Joyce AR Reed JL White A Edwards R Osterman A Baba T Mori H Lesely SA Palsson BO Agarwalla S Experimental and computational assessment of conditionally essential genes in Escherichia coli J Bacteriol 2006 188 8259 8271 10.1128/JB.00740-06 17012394 PMC1698209

[B35] Barve A Rodrigues JF Wagner A Superessential reactions in metabolic networks Proc Natl Acad Sci U S A 2012 109 E1121 E1130 10.1073/pnas.1113065109 22509034 PMC3345022

[B36] Glasner JD Rusch M Liss P Plunkett G 3rd Cabot EL Darling A Anderson BD Infield-Harm P Gilson MC Perna NT ASAP: a resource for annotating, curating, comparing, and disseminating genomic data Nucleic Acids Res 2006 34 D41 45 10.1093/nar/gkj164 16381899 PMC1347526

[B37] Schwartz AS Pachter L Multiple alignment by sequence annealing Bioinformatics 2007 23 e24 29 10.1093/bioinformatics/btl311 17237099

[B38] Felsenstein J PHYLIP - Phylogeny Inference Package (Version 3.2) Cladistics - the International J of the Wellihennig Soc 1989 5 164 166

[B39] Felsenstein J PHYLIP (Phylogeny Inference Package) version 3.6. Distributed by the author. Department of Genome Sciences 2005 Washington: University of Washington

[B40] Guindon S Dufayard JF Lefort V Anisimova M Hordijk W Gascuel O New algorithms and methods to estimate maximum-likelihood phylogenies: assessing the performance of PhyML 3.0 Syst Biol 2005 59 307 321 20525638 10.1093/sysbio/syq010

[B41] Gascuel O BIONJ: an improved version of the NJ algorithm based on a simple model of sequence data Mol Biol Evol 1997 14 685 695 10.1093/oxfordjournals.molbev.a025808 9254330

[B42] Whelan S Goldman N A general empirical model of protein evolution derived from multiple protein families using a maximum-likelihood approach Mol Biol Evol 2001 18 691 699 10.1093/oxfordjournals.molbev.a003851 11319253

[B43] Orth JD Thiele I Palsson BO What is flux balance analysis? Nat Biotechnol 2010 28 245 248 10.1038/nbt.1614 20212490 PMC3108565

[B44] Feist AM Palsson BO The biomass objective function Curr Opin Microbiol 2010 13 344 349 10.1016/j.mib.2010.03.003 20430689 PMC2912156

[B45] Kang Y Weber KD Qiu Y Kiley PJ Blattner FR Genome-wide expression analysis indicates that FNR of Escherichia coli K-12 regulates a large number of genes of unknown function J Bacteriol 2005 187 1135 1160 10.1128/JB.187.3.1135-1160.2005 15659690 PMC545700

[B46] Perna NT Plunkett G 3rd Burland V Mau B Glasner JD Rose DJ Mayhew GF Evans PS Gregor J Kirkpatrick HA Posfai G Hackett J Klink S Boutin A Shao Y Miller L Grotbeck EJ Davis NW Lim A Dimalanta ET Potamousis KD Apodaca J Anantharaman TS Lin J Yen G Schwartz DC Welch RA Blattner FR Genome sequence of enterohaemorrhagic Escherichia coli O157:H7 Nature 2001 409 529 533 10.1038/35054089 11206551

[B47] Hayashi T Makino K Ohnishi M Kurokawa K Ishii K Yokoyama K Han CG Ohtsubo E Nakayama K Murata T Tanaka M Tobe T Iida T Takami H Honda T Sasakawa C Ogasawara N Yasunaga T Kuhara S Shiba T Hattori M Shinagawa H Complete genome sequence of enterohemorrhagic Escherichia coli O157:H7 and genomic comparison with a laboratory strain K-12 DNA Res 2001 8 11 22 10.1093/dnares/8.1.11 11258796

[B48] Welch RA Burland V Plunkett G 3rd Redford P Roesch P Rasko D Buckles EL Liou SR Boutin A Hackett J Stroud D Mayhew GF Rose DJ Zhou S Schwartz DC Perna NT Mobley HL Donnenberg MS Blattner FR Extensive mosaic structure revealed by the complete genome sequence of uropathogenic Escherichia coli Proc Natl Acad Sci U S A 2002 99 17020 17024 10.1073/pnas.252529799 12471157 PMC139262

[B49] Chen SL Hung CS Xu J Reigstad CS Magrini V Sabo A Blasiar D Bieri T Meyer RR Ozersky P Armstrong JR Fulton RS Latreille JP Spieth J Hooton TM Mardis ER Hultgren SJ Gordon JI Identification of genes subject to positive selection in uropathogenic strains of Escherichia coli: a comparative genomics approach Proc Natl Acad Sci U S A 2006 103 5977 5982 10.1073/pnas.0600938103 16585510 PMC1424661

[B50] Wei J Goldberg MB Burland V Venkatesan MM Deng W Fournier G Mayhew GF Plunkett G 3rd Rose DJ Darling A Mau B Perna NT Payne SM Runyen-Janecky LJ Zhou S Schwartz DC Blattner FR Complete genome sequence and comparative genomics of Shigella flexneri serotype 2a strain 2457T Infect Immun 2003 71 2775 2786 10.1128/IAI.71.5.2775-2786.2003 12704152 PMC153260

[B51] Boyd JM Lewis JA Escalante-Semerena JC Downs DM Salmonella enterica requires ApbC function for growth on tricarballylate: evidence of functional redundancy between ApbC and IscU J Bacteriol 2008 190 4596 4602 10.1128/JB.00262-08 18441067 PMC2446783

[B52] Sebaihia M Bocsanczy AM Biehl BS Quail MA Perna NT Glasner JD DeClerck GA Cartinhour S Schneider DJ Bentley SD Parkhill J Beer SV Complete genome sequence of the plant pathogen Erwinia amylovora strain ATCC 49946 J Bacteriol 2010 192 2020 2021 10.1128/JB.00022-10 20118253 PMC2838050

[B53] Babujee L Apodaca J Balakrishnan V Liss P Kiley PJ Charkowski AO Glasner JD Perna NT Evolution of the metabolic and regulatory networks associated with oxygen availability in two phytopathogenic enterobacteria BMC Genomics 2012 13 110 10.1186/1471-2164-13-110 22439737 PMC3349551

