# Supplementary figures and images for: Inferring ancient metabolism using ancestral core metabolic models of enterobacteria
Source: BMC Syst Biol. 2013 Jun 11;7:46. doi: 10.1186/1752-0509-7-46 (PMC3694032; doi:10.1186/1752-0509-7-46)

Additional File 1.

Aerobic

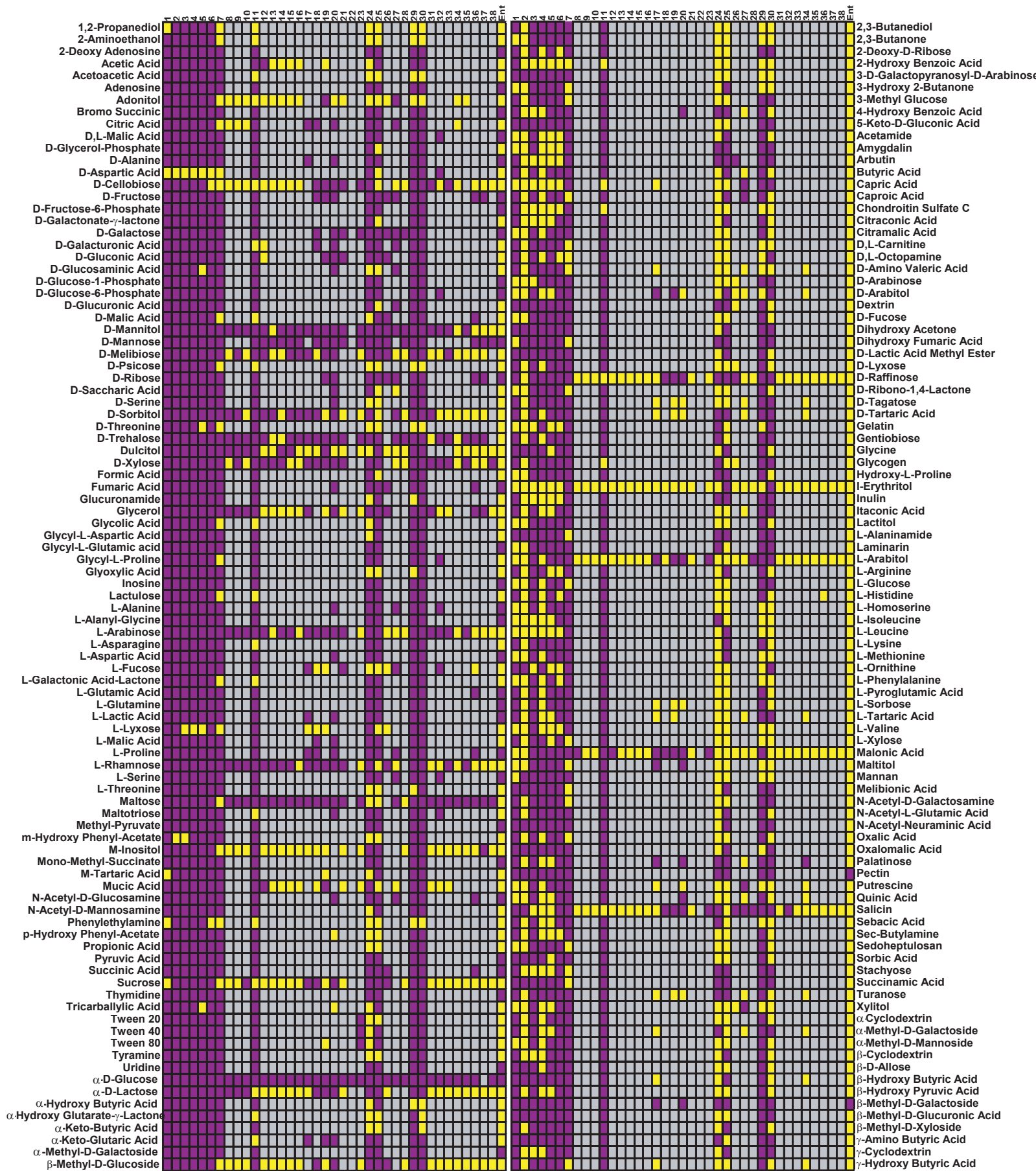

Yellow = No growth    Purple = Yes growth    Grey = Not determined

Supplement: Additional file 1 — Aerobic experimental carbon source utilization for 38 enterobacterial strains. [file 1752-0509-7-46-S1.pdf]

Supplementary Figure 2. Anaerobic

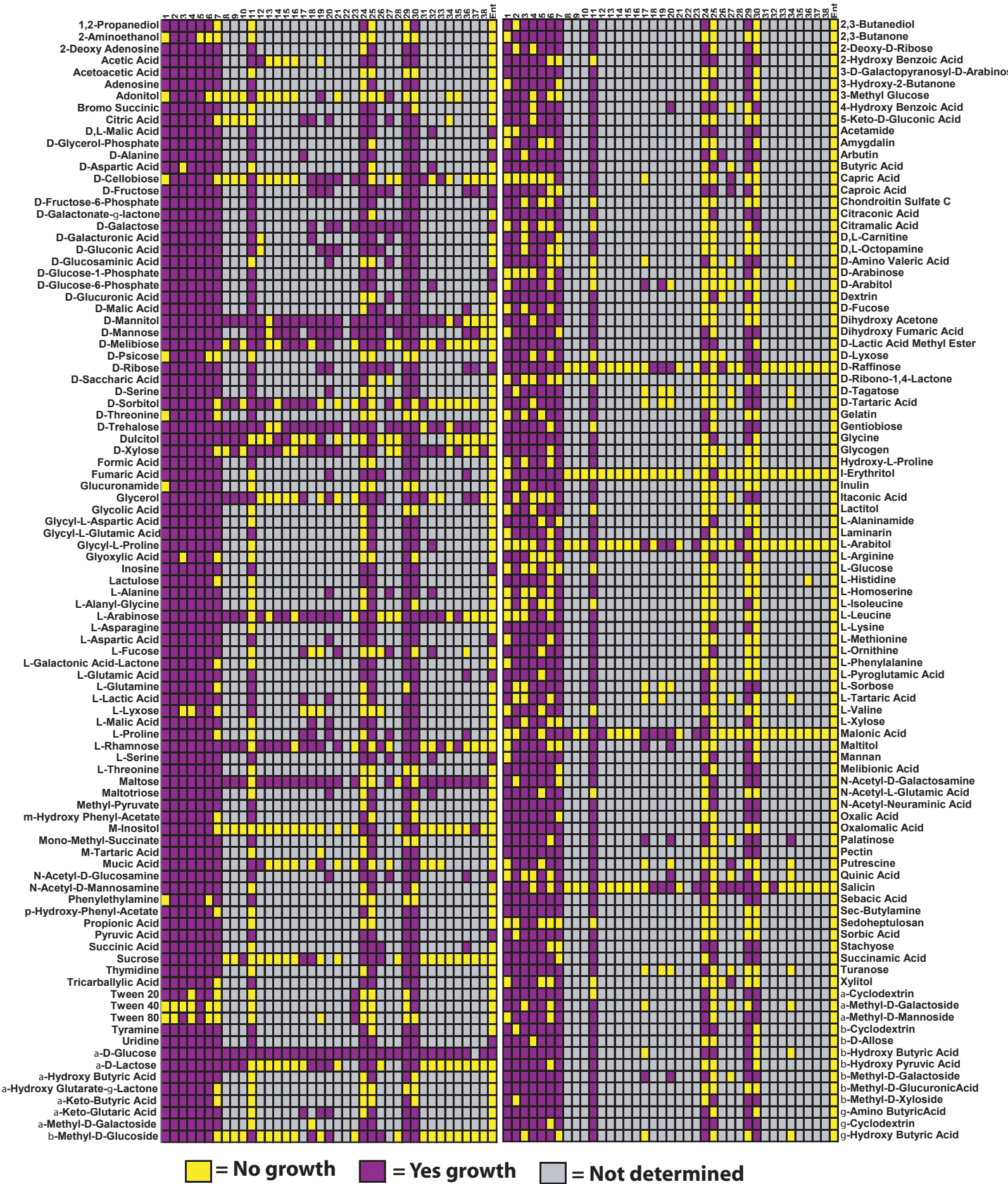

Supplement: Additional file 2 — Anaerobic experimental carbon source utilization for 38 enterobacterial strains. [file 1752-0509-7-46-S2.pdf]
