# Supplementary material for: Inferring ancient metabolism using ancestral core metabolic models of enterobacteria
Source: BMC Syst Biol. 2013 Jun 11;7:46. doi: 10.1186/1752-0509-7-46 (PMC3694032; doi:10.1186/1752-0509-7-46)
Supplement: Additional file 4: Table S1 — List of genomes used for the phylogenetic analysis of the enterobacteria. [file 1752-0509-7-46-S4.doc]

| **Genus** | **Type species** | **Type strain** | **Strain used in this study** | **Accession #** | **Ref.** |
| --- | --- | --- | --- | --- | --- |
| *Brenneria* | Brenneria salicis | ATCC 15712 | Same as Type strain | private | 1 |
| *Cedecea* | *Cedecea davisae* | ATCC 33431 | Same as Type strain | private | 2 |
| *Citrobacter* | *Citrobacter freundii* | ATCC 8090 | *Citrobacter koseri* ATCC BAA-895 | CP000822.1 | 3,4 |
| *Cronobacter* | *Cronobacter sakazakii* | ATCC 29544 | *Cronobacter sakazakii* ATCC BAA-894 | CP000783.1 | 5,6 |
| *Dickeya* | *Dickeya chrysanthemi* | ATCC 11663 | Dickeya dadantii 3937 | CP002038.1 | 7 |
| *Edwardsiella* | *Edwardsiella tarda* | ATCC 15974 | *Edwardsiella tarda* EIB202 | CP001135.1 | 3,8 |
| *Enterobacter* | *Enterobacter cloacae* | ATCC 13047 | Same as Type strain | CP001918.1 | 9 |
| *Erwinia* | *Erwinia amylovora* | ATCC 15580 | *Erwinia amylovora* ATCC 49946 | FN666575.1 | 3,10 |
| *Escherichia* | *Escherichia coli* | ATCC 11775 | *Escherichia coli* K-12 MG1655 | U00096.2 | 3,11 |
| *Ewingella* | *Ewingella americana* | ATCC 33852 | Same as Type strain | private | 12 |
| *Hafnia* | *Hafnia alvei* | ATCC 13337 | Same as Type strain | private | 3 |
| *Klebsiella* | *Klebsiella pneumoniae* | ATCC 13883 | *Klebsiella pneumoniae* NTUH-K2044 | AP006725.1 | 3,13 |
| *Leclercia* | *Leclercia adecarboxylata* | ATCC 23216 | Same as Type strain | private | 14 |
| *Pantoea* | *Pantoea agglomerans* | ATCC 27155 | *Pantoea ananatis* LMG 20103 | CP001875.1 | 15,16 |
| *Pectobacterium* | *Pectobacterium carotovorum* | ATCC 15713 | *Pectobacterium carotovorum subsp. carotovorum PC1* | CP001657.1 | 3 |
| *Photorhabdus* | *Photorhabdus luminescens* | ATCC 29999 | *Photorhabdus luminescens subsp. laumondii* TTO1 | BX470251.1 | 17,18 |
| *Proteus* | *Proteus vulgaris* | ATCC 13315 | *Proteus mirabilis* HI4320 | AM942759.1 | 3,19 |
| *Salmonella* | *Salmonella enterica* | LT2 | Same as Type strain | AE006468.1 | 20,21 |
| *Serratia* | *Serratia marcescens* | ATCC 13880 | Same as Type strain | private | 3 |
| *Shigella* | *Shigella dysenteriae* | ATCC 13313 | *Shigella dysenteriae* Sd197 | CP000034.1 | 3,22 |
| *Xenorhabdus* | *Xenorhabdus nematophila* | ATCC 19061 | Same as Type strain | private | 3 |
| *Yersinia* | *Yersinia pestis* | ATCC 19428 | *Yersinia pestis* CO92 | AL590842.1 | 3,23 |
| *Yokenella* | *Yokenella regensburgei* | ATCC 49455 | Same as Type strain | private | 24 |

**Additional file 3**

**References for the genomes listed above**

1 Hauben, L. *et al.* Phylogenetic position of phytopathogens within the Enterobacteriaceae. *Systematic and Applied Microbiology* 21, 384-397 (1998).

2 Grimont, P. A. D., Grimont, F., Farmer, J. J. & Asbury, M. A. Cedecea-Davisae Gen-Nov, Sp-Nov and Cedecea-Lapagei Sp-Nov, New Enterobacteriaceae from Clinical Specimens. *International Journal of Systematic Bacteriology* 31, 317-326 (1981).

3 Skerman, V. B. D., Mcgowan, V. & Sneath, P. H. A. Approved Lists of Bacterial Names. *International Journal of Systematic Bacteriology* 30, 225-420 (1980).

4 Brenner, D. J. *et al.* Classification of Citrobacteria by DNA Hybridization - Designation of Citrobacter-Farmeri Sp-Nov, Citrobacter-Youngae Sp-Nov, Citrobacter-Braakii Sp-Nov, Citrobacter-Werkmanii Sp-Nov, Citrobacter-Sedlakii Sp-Nov, and 3 Unnamed Citrobacter Genomospecies. *International Journal of Systematic Bacteriology* 43, 645-658 (1993).

5 Kucerova, E. *et al.* Genome Sequence of Cronobacter sakazakii BAA-894 and Comparative Genomic Hybridization Analysis with Other Cronobacter Species. *Plos One* 5, A51-A60 (2010).

6 Iversen, C. *et al.* Cronobacter gen nov, a new genus to accommodate the biogroups of Enterobacter sakazakii, and proposal of Cronobacter sakazakii gen nov, comb nov, Cronobacter malonaticus sp nov, Cronobacter turicensis sp nov, Cronobacter muytjensii sp nov, Cronobacter dublinensis sp nov, Cronobacter genomospecies 1, and of three subspecies, Cronobacter dublinensis subsp dublinensis subsp nov, Cronobacter dublinensis subsp lausannensis subsp nov and Cronobacter dublinensis subsp lactaridi subsp nov. *International Journal of Systematic and Evolutionary Microbiology* 58, 1442-1447 (2008).

7 Samson, R. *et al.* Transfer of *Pectobacterium chrysanthemi* (Burkholder et al. 1953) Brenner et al. 1973 and *Brenneria paradisiaca* to the genus *Dickeya* gen. nov as *Dickeya chrysanthemi* comb. nov and *Dickeya paradisiaca* comb. nov and delineation of four novel species, *Dickeya dadantii* sp nov., *Dickeya dianthicola* sp nov., *Dickeya dieffenbachiae* sp nov and *Dickeya zeae* sp nov. *International Journal of Systematic and Evolutionary Microbiology* 55, 1415-1427 (2005).

8 W.H. EWING, A. C. M., M R ESCOBAR and A.H. LUBIN. Edwardsiella, a new genus of Enterobacteriaceae based on a new species, E. tarda. *International Bulletin of Bacteriological Nomenclature and Taxonomy*, 33-38 (1965).

9 Hoffmann, H. *et al.* Reassignment of Enterobacter dissolvens to Enterobacter cloacae as E-cloacae subspecies dissolvens comb. nov and emended description of Enterobacter asburiae and Enterobacter kobei. *Systematic and Applied Microbiology* 28, 196-205 (2005).

10 Sebaihia, M. *et al.* Complete genome sequence of the plant pathogen Erwinia amylovora strain ATCC 49946. *J Bacteriol* 192, 2020-2021.

11 Blattner, F. R. *et al.* The complete genome sequence of Escherichia coli K-12. *Science* 277, 1453-1462 (1997).

12 Grimont, P. A. *et al.* Ewingella americana gen.nov., sp.nov., a new Enterobacteriaceae isolated from clinical specimens. *Ann Microbiol (Paris)* 134A, 39-52 (1983).

13 Wu, K. M. *et al.* Genome Sequencing and Comparative Analysis of Klebsiella pneumoniae NTUH-K2044, a Strain Causing Liver Abscess and Meningitis. *Journal of Bacteriology* 191, 4492-4501 (2009).

14 Tamura, K., Sakazaki, R., Kosako, Y. & Yoshizaki, E. Leclercia-Adecarboxylata Gen-Nov, Comb-Nov, Formerly Known as Escherichia-Adecarboxylata. *Current Microbiology* 13, 179-184 (1986).

15 Gavini, F. *et al.* Transfer of Enterobacter-Agglomerans (Beijerinck 1888) Ewing and Fife 1972 to Pantoea Gen-Nov as Pantoea-Agglomerans Comb Nov and Description of Pantoea-Dispersa Sp-Nov. *International Journal of Systematic Bacteriology* 39, 337-345 (1989).

16 De Maayer, P. *et al.* Genome Sequence of Pantoea ananatis LMG20103, the Causative Agent of Eucalyptus Blight and Dieback. *Journal of Bacteriology* 192, 2936-2937 (2010).

17 Duchaud, E. *et al.* The genome sequence of the entomopathogenic bacterium Photorhabdus luminescens. *Nature Biotechnology* 21, 1307-1313 (2003).

18 Boemare, N. E., Akhurst, R. J. & Mourant, R. G. DNA Relatedness between Xenorhabdus Spp (Enterobacteriaceae), Symbiotic Bacteria of Entomopathogenic Nematodes, and a Proposal to Transfer Xenorhabdus-Luminescens to a New Genus, Photorhabdus Gen-Nov. *International Journal of Systematic Bacteriology* 43, 249-255 (1993).

19 Pearson, M. M. *et al.* Complete genome sequence of uropathogenic Proteus mirabilis, a master of both adherence and motility. *Journal of Bacteriology* 190, 4027-4037 (2008).

20 Prokaryotes, J. C. o. t. I. C. o. S. o. The type species of the genus Salmonella Lignieres 1900 is Salmonella enterica (ex Kauffmann and Edwards 1952) Le Minor and Popoff 1987, with the type strain LT2T, and conservation of the epithet enterica in Salmonella enterica over all earlier epithets that may be applied to this species. Opinion 80. *Int J Syst Evol Microbiol* 55, 519-520 (2005).

21 McClelland, M. *et al.* Complete genome sequence of Salmonella enterica serovar Typhimurium LT2. *Nature* 413, 852-856 (2001).

22 Yang, F. *et al.* Genome dynamics and diversity of Shigella species, the etiologic agents of bacillary dysentery. *Nucleic Acids Res* 33, 6445-6458 (2005).

23 Parkhill, J. *et al.* Genome sequence of Yersinia pestis, the causative agent of plague. *Nature* 413, 523-527 (2001).

24 Kosako, Y., Sakazaki, R. & Yoshizaki, E. Yokenella-Regensburgei Gen-Nov, Sp-Nov - a New Genus and Species in the Family Enterobacteriaceae. *Japanese Journal of Medical Science & Biology* 37, 117-124 (1984).

25 Munson, M. A., Baumann, P. & Kinsey, M. G. Buchnera Gen-Nov and Buchnera-Aphidicola Sp-Nov, a Taxon Consisting of the Mycetocyte-Associated, Primary Endosymbionts of Aphids. *International Journal of Systematic Bacteriology* 41, 566-568 (1991).

26 Aksoy, S. Wigglesworthia Gen-Nov and Wigglesworthia-Glossinidia Sp-Nov, Taxa Consisting of the Mycetocyte-Associated, Primary Endosymbionts of Tsetse-Flies. *International Journal of Systematic Bacteriology* 45, 848-851 (1995).

27 Dale, C. & Maudlin, I. Sodalis gen. nov. and Sodalis glossinidius sp. nov., a microaerophilic secondary endosymbiont of the tsetse fly Glossina morsitans morsitans. *International Journal of Systematic Bacteriology* 49, 267-275 (1999).
